# Lithium management in pregnant patients with bipolar disorder

**DOI:** 10.1192/j.eurpsy.2023.467

**Published:** 2023-07-19

**Authors:** I. Romero Gerechter, M. Martín Velasco, A. Sanz Giancola, E. Arroyo Sánchez, C. Díaz Mayoral, P. Setien Preciados

**Affiliations:** Psychiatry, Hospital Universitario Príncipe de Asturias, Madrid, Spain

## Abstract

**Introduction:**

Women with bipolar disorder often ask their treating clinician for information about family planning, as they are concerned about the impact of their illness on offspring. Pregnancy places additional stress on patients, and physiological changes are particularly acute during postpartum. On the other hand, the risk of abnormalities and teratogenicity from psychotropic drugs is significant. The decision wether resuming or discontinuating lithium is discussed.

**Objectives:**

We present a theoretical review on the topic.

**Methods:**

A bibliographic review is presented.

**Results:**

The choice to continue medication during pregnancy balances the risks of an untreated illness with the risks of medication exposure. Abrupt discontinuation of psychotropic medications is associated with an increased risk for illness recurrence. Women with BD who discontinue their medications before or during pregnancy have a 71% risk of recurrence with new episodes occurring most frequently in the first trimester. Recurrent illness during pregnancy is associated with a 66% increase in the risk of postpartum episodes. Untreated or under-treated BD during pregnancy is associated with poor birth outcomes independent of pharmacotherapy exposure, including preterm birth, low birth-weight, intrauterine growth retardation, small for gestational age, fetal distress, and adverse neurodeve- lopmental outcomes. Women with untreated BD also have behavioral risk factors such as decreased compliance with prenatal care, poor nutrition, and high-risk behaviors. Impaired capacity to function may result in loss of employment, health care benefits, and social support. The biological and psychosocial risks of a BD episode are the justification for the risk of medication exposure.

Fetal exposure to lithium has been associated with an increased risk for cardiac abnormalities. The risk for Ebstein’s anomaly with first trimester exposure is 1 (0.1%) to 2 in 1000 (0.2%), but the absolute risk remains low. Folate supplementation with 5 mg reduces the risk and severity of congenital heart disease. Lithium toxicity causes lethargy, hypotonia, tachycardia, coma, cyanosis, and chronic twitching in the newborn.

Strategies to minimize fetal exposure and maintain efficacy include using the lowest effective dose, prescribing lithium twice daily to avoid high peak serum concentrations, and regular monitoring of lithium serum concentrations. The effective serum concentration must be established before pregnancy. If a therapeutic concentration has not been established, the lithium dose is titrated to a concentration within the therapeutic range. Breast feeding is discouraged in women taking lithium because of the high rate of transmission to the infant.

**Conclusions:**

Treatment decisions for pregnant women with mood disorders must weigh the potential for increased risks of lithium during pregnancy, especially during the first trimester, against its effectiveness at reducing relapse.

**Disclosure of Interest:**

None Declared

